# Enteric Nervous System: The Bridge Between the Gut Microbiota and Neurological Disorders

**DOI:** 10.3389/fnagi.2022.810483

**Published:** 2022-04-19

**Authors:** Zi-Han Geng, Yan Zhu, Quan-Lin Li, Chao Zhao, Ping-Hong Zhou

**Affiliations:** ^1^Endoscopy Center and Endoscopy Research Institute, Zhongshan Hospital, Fudan University, Shanghai, China; ^2^Shanghai Collaborative Innovation Center of Endoscopy, Shanghai, China; ^3^Key Laboratory of Medical Molecular Virology, School of Basic Medical Sciences & National Clinical Research Center for Aging and Medicine, Huashan Hospital, Fudan University, Shanghai, China

**Keywords:** gastrointestinal microbiota, enteric nervous system, central nervous system, neurological disorders, microbiota-gut-brain axis

## Abstract

The gastrointestinal (GI) tract plays an essential role in food digestion, absorption, and the mucosal immune system; it is also inhabited by a huge range of microbes. The GI tract is densely innervated by a network of 200–600 million neurons that comprise the enteric nervous system (ENS). This system cooperates with intestinal microbes, the intestinal immune system, and endocrine systems; it forms a complex network that is required to maintain a stable intestinal microenvironment. Understanding how gut microbes influence the ENS and central nervous system (CNS) has been a significant research subject over the past decade. Moreover, accumulating evidence from animal and clinical studies has revealed that gut microbiota play important roles in various neurological diseases. However, the causal relationship between microbial changes and neurological disorders currently remains unproven. This review aims to summarize the possible contributions of GI microbiota to the ENS and CNS. It also provides new insights into furthering our current understanding of neurological disorders.

## Introduction

Incidences of neurological disorders gradually increase with age in humans, accompanied by poor prognoses and associated social burdens. Drokhlyansky et al. (2020) found that disease-related genes were dysregulated with aging, and that the enteric nervous system (ENS) expressed risk genes for neuropathic, inflammatory, and extraintestinal diseases. This suggests that there are neuronal contributions to such diseases (Drokhlyansky et al., 2020). The microbiota-gut-brain (MGB) axis provides a new understanding of the essence of these diseases; however, to date research has been limited to analysis of the correlations between microbiota and different mechanisms; it lacks support from more favorable evidence.

The gastrointestinal (GI) tract harbors a considerable number of commensal microorganisms, including bacteria, viruses, fungi, and protozoans, which frequently vary depending on the host’s diet, drug intervention, and diseases. The bacteria in the GI tract are relatively stable; they comprise four main phyla: Bacteroidetes, Firmicutes, Actinobacteria, and Proteobacteria. The ENS can be reasonably regarded as the bridge between the intestinal microbiota and the nervous system. The ENS is considered to be a separate branch of the autonomic nervous system (ANS); it is located throughout the length of the GI tract and is arranged into two ganglionated plexuses: the submucosal plexus (Meissner’s plexus) and the myenteric plexus (Auerbach’s Plexus), which comprise nitrergic and cholinergic enteric neurons. The ENS also hosts an abundance of calretinin- or neuropeptide-expressing neurons, as well as catecholaminergic and inhibitory gamma-aminobutyric acid (GABA) neurons (Qu et al., 2008). The ENS forms a complete sensorimotor reflex circuit consisting of intrinsic primary afferent neurons (IPANs), interneurons, and motor neurons within the gut wall; this circuit is responsible for the coordination of gut functions such as motility, peristalsis, and intestinal mucosal immunity (Wehrwein et al., 2016; Allaire et al., 2018). Interestingly, Smith-Edwards et al. (2021) found that sympathetic neurons can regulate the activity of neurons and non-neuronal cells differentially in the proximal and distal colon, thereby promoting distinct changes in motility patterns.

Given the proximity of the GI microbiota to the ENS, gut microbes can influence its development and function—either directly or indirectly. These complex interactions involve signaling that is initiated by microbe- or host-derived components or metabolites; they eventually affect enteric nervous excitability and GI function.

The cell bodies of the sensory vagal fibers that relay sensory information from the ENS to the central nervous system (CNS) reside in the nodose/jugular ganglia and the dorsal root ganglia (DRG). These vagal fibers mostly synapse bilaterally on the nucleus tractus solitarius (NTS). The individual components of the MGB axis communicate with each other bi-directionally (either antagonistically or synergistically) within the ANS. The ANS, in combination with the hypothalamic-pituitary-adrenal (HPA) axis and neuroendocrine signaling, can induce CNS-modulated changes in the gut (Mayer et al., 2015). The enteric immune and enteric endocrine systems also influence ENS activity and neurochemistry.

The GI tract incessantly monitors the dynamic intestinal microenvironment, where enteroendocrine and immune cells can facilitate signaling from the gut lumen to the ENS. During this process, neuropods have been proposed to transduce signals from enteroendocrine cells to sensory neurons (Bohórquez et al., 2015). Enteric neurons, mucosal immune cells [e.g., muscularis macrophages (MMs)], 5-hydroxytryptamine (5-HT) from enteroendocrine cells, molecules from gut microbes [e.g., lipopolysaccharides (LPSs) and short chain fatty acids (SCFAs)], and diet ingredients, all contribute to gut sensation, secretion, and motility.

As a bidirectional link, the ENS connects the GI microbiota with many of the host’s systems. It is widely believed that the gut microbiota are critically important for the appropriate development and maintenance of ENS function. Given that disorders of the ENS correlate with CNS diseases, exploring the mechanisms of gut microbe-ENS interactions could provide a new perspective for furthering our understanding of neurological disorders; it could also help develop novel therapeutic strategies.

## Influence of GI Microbiota on the ENS

The gut exposes the host to the external environment; it has a mucosal surface of approximately 32 m^2^ (Helander and Fändriks, 2014) and interfaces intimately with commensal bacteria and the molecules they produce, including microbe-derived components and metabolites such as microbe-associated molecular patterns (MAMPs), neurotransmitters, and hormones. A general reduction in the myenteric plexus density has been observed in both the jejunum and the ileum of postnatal germ-free (GF) mice, but not in the duodenum; this suggests that early exposure to gut microbes is essential for the postnatal development of the ENS (Collins et al., 2014).

### Direct Interactions Between GI Microbiota and the ENS

Most commensal microbes are separated from the gut epithelial surface by a layer of mucin; hence, they are unlikely to directly interact with the GI tissue. Furthermore, very few gut bacteria directly contact the epithelium or live in the adherent mucus layer (Derrien et al., 2011; Johansson et al., 2011). Therefore, there are several methods of communication between bacteria and their hosts, including the production of molecules as a consequence of the degradation of microbiota constituents and the synthesis and secretion of molecules by bacteria.

#### Microbe-Derived Components

Microbe-associated molecular patterns are molecular signatures that are highly conserved across whole classes of microbes but are absent in the host, such as LPS or polysaccharide A (PSA) on the surface of gram-negative bacteria or peptidoglycan (PGN) on the surface of gram-positive bacteria (Medzhitov, 2007; Boller and Felix, 2009). Each MAMP is detected by specific surface-localized receptors that are termed pattern recognition receptors (PRRs), such as transmembrane, surface, or endosome toll-like receptors (TLRs); they are expressed in myenteric neurons, enteric glial cells (EGCs), and innate immune cells.

TLR3 and TLR7, which recognize viral RNA, and TLR4, which recognizes LPS, have been demonstrated in the ENS of the murine intestine and human ileum, both of which innervate Peyer’s patches. Moreover, TLR4 is abundantly expressed in the murine distal large bowel (Barajon et al., 2009; Boller and Felix, 2009), while TLR4–/– mice are characterized by a reduced number of nitrergic inhibitory neurons and abnormal intestinal motility. These characteristics have also been observed in GF mice, antibiotics-treated mice, and mice with the ENS-specific deletion of myeloid differentiation primary response 88 (MyD88) (Anitha et al., 2012; Hung et al., 2019, 2020). MyD88, as an adaptor molecule, is essential for TLR-mediated signal transduction. TLR4 is also expressed in enteric stem cell niches, and intense interplay has been observed between LPS and Wnt signaling during enteric stem cell regulation (Schuster et al., 2014; Di Liddo et al., 2015). Therefore, the LPS-mediated activation of TLR4 appears to promote the survival of enteric neurons and regulate gut motility (Anitha et al., 2012), suggesting that the TLR4 pathway plays a critical role in the development of ENS.

TLR2 is expressed in the enteric neurons, EGCs, and smooth muscle cells of the intestinal wall; it detects gram-negative bacterial PSA and gram-positive bacterial PGN and lipoprotein. A phenotype similar to that observed in TLR4–/– mice, the reduction of nitrergic neurons, and acetylcholine-esterase (AChE) fibers in the myenteric ganglia have all been observed in TLR2–/– mice, accompanied by gut dysmotility (Brun et al., 2013). Carbachol has been shown to induce a strong tetrodotoxin-dependent chloride secretion, as shown by short-circuit current increment in wild type (WT) mice, but not in TLR2–/– mice (Brun et al., 2013). Furthermore, TLR2 signaling has been demonstrated to affect the composition of neurons in the ENS and intestinal contractility (Brun et al., 2013).

These observations show that TLR pathways allow microbes to access the ENS. Thus, bacterial or viral components may directly activate the ENS. Nevertheless, the mechanisms by which TLR ligands control enteric neurogenesis require further investigation.

#### Microbe-Derived Metabolites

The most extensively studied gut microbe-derived metabolites are SCFAs, which are bacterial fermentation products that originate from starch and fiber; more than 95% of SCFAs consist of acetate, propionate, and butyrate. A wide range of pre-clinical evidence supports the involvement of SCFAs as modulators of not only colonic function but also multiple inflammatory and metabolic processes (Erny et al., 2017; Gill et al., 2018; Krautkramer et al., 2020). SCFAs also majorly contribute directly to the integrity of the intestinal epithelial barrier by regulating mucus production and the expression of tight junction proteins. Therefore, SCFAs have been implicated as the first line of defense between gut microbes and the permeability of the host’s intestinal barrier.

Short chain fatty acids can activate several G protein-coupled receptors (GPCRs), of which free fatty acid receptors 2 and 3 (FFAR2 and FFAR3, also referred to as GPR43 and GPR41, respectively) have been studied the most extensively. SCFAs can activate the peripheral nervous system (PNS), in which FFAR3 has been detected in the enteric neural plexus, autonomic and somatic sensory ganglia, and portal nerve (Nøhr et al., 2015; Kaji et al., 2016). In the gut, FFAR3 is expressed on nitrergic and cholinergic enteric neurons.

All SCFAs inhibit the activity of histone deacetylases (HDACs); butyrate has been reported to present the most potent activity (Cleophas et al., 2016). Furthermore, acetate can be converted to acetyl-CoA, which increases the acetylation of histone (Soliman et al., 2012). SCFAs have also been shown to influence the neurochemical phenotype of the ENS of an adult rat. Butyrate can increase the proportion of myenteric cholinergic neurons by inhibiting HDACs but not that of nitrergic neurons. Both the application of butyrate and the adoption of a resistant starch diet (RSD) have been found to increase the colonic concentrations of butyrate and enhance gut motility (Soret et al., 2010). Therefore, SCFAs might be involved in maintaining ENS homeostasis.

Polyamines are metabolites of gut microbes that play essential roles in stress responses, inflammation, and neuronal signaling in the CNS and ENS. Moreover, polyamines are required for the expression of TLR2; hence, they modulate the integrity of the intestinal epithelial barrier (Chen et al., 2007).

#### Microbe-Derived Neurochemicals

Microbial species can produce an array of neurotransmitters, such as GABA, norepinephrine (NE), 5-HT, dopamine, and acetylcholine. They can target intestinal and extraintestinal immune/neuronal elements. Therefore, the study of microbe-derived neurochemicals, a field referred to as “Microbial endocrinology” (Lyte, 2011, 2014), is becoming essential for advancing our knowledge of interactions between gut microbes and the ENS. This suggests that the bacterial production of neurotransmitters may be a primary molecular mechanism of bacteria-neuron communication.

### Indirect Interactions Between GI Microbiota and the ENS

Bacterial products such as MAMPs, SCFAs, and microvesicles (MVs) interact with the enteric immune system, enteric epithelium, smooth muscle, and endocrine system. All of these systems contribute to the maintenance of intestinal immune innate tolerance, the development of the adaptive immune system, and the homeostasis of the intestinal microenvironment. In turn, molecular mediators that are secreted by gut-resident immune cells and enteroendocrine cells in the intestinal epithelium can be detected by corresponding receptors in the ENS; they can also affect enteric function (Kamada et al., 2013; La Fata et al., 2018). Moreover, intestinal stromal cells, such as interstitial cells of Cajal (ICCs) and EGCs, might participate in the interactions between gut microbes and the ENS.

#### Enteric Immune System

Gastrointestinal microorganisms activate resident immune cells within the gut; they may signal to enteric neurons through immune mediators (Maynard et al., 2012; Sampson and Mazmanian, 2015; Rooks and Garrett, 2016). Indeed, the dysbiosis of intestinal flora, uncontrolled immune responses to pathogenic stimuli, and adaptive changes in the ENS represent the main factors in the pathogenesis of several GI disorders (Vindigni et al., 2016; Zhang et al., 2017).

A distinct population of macrophages that is distributed throughout the intestinal muscularis externa regulates the peristaltic activity of the colon under a steady state. MMs display a tissue-protective phenotype, whereas lamina propria macrophages (LpMs) preferentially express a pro-inflammatory phenotype. MMs express high levels of Adrb2 [Adrenoceptor Beta 2, which encodes b2 adrenergic receptors (ARs)], which is the most significantly differentially expressed gene between MM and LpM populations (Chang et al., 2014). Adrb2 is essential for NE signaling and MMs reside close to enteric neurons labeled with the calcium indicator GCaMP3. MMs have been suggested to interact with active neurons in the gut muscularis. MMs change the patterns of smooth muscle contractions by secreting bone morphogenetic protein 2 (BMP2), which activates the BMP receptor (BMPR) that is expressed by enteric neurons. In turn, enteric neurons secrete colony stimulatory factor1 (CSF1), which is required for the development of MMs as a growth factor. Finally, LPS from gut microbes regulate the expression of BMP2 by MMs and the expression of CSF1 by enteric neurons. Therefore, the cross-talk between the ENS and the muscular macrophages is dependent on LPS from intestinal bacteria (Muller et al., 2014; Gabanyi et al., 2016).

In addition to being expressed in enteric neurons, EGCs, and the smooth muscle cells of the intestinal wall, TLR2 is also expressed in dendritic cells (DCs) (Wang et al., 2006). As an immunodominant component of the *Bacteroides fragilis* capsule, PSA can interact with TLR2 on DCs. The application of live *B. fragilis* or pure PSA can evoke action potentials in IPANs within approximately 5 s. This leads to an increased excitability of IPANS. However, mutant *B. fragilis* that are devoid of PSA were observed to fail to elicit such a response regarding the activity of IPANs (Mao et al., 2013). In contrast, LPS were not observed to affect the activity of IPANs. Based on the latency of the neuronal response, PSA may first act on gut-resident immune cells, which would then activate IPANs via the release of an intermediate mediator (Ochoa-Repáraz et al., 2010; Mao et al., 2013).

As noted previously, SCFAs could prevent the activity of IPANs from affecting the host’s gene expression. The inhibition of HDACs enhances the histone acetylation of gene regulatory elements and increases gene transcription (Falkenberg and Johnstone, 2014). Epigenetic regulation by butyrate has been demonstrated in colonic T cells and macrophages within the immune system (Arpaia et al., 2013; Furusawa et al., 2013; Chang et al., 2014). In contrast, however, little is known about the epigenetic modification of ENS by SCFAs. In the future, therefore, it is essential to determine which microbe-derived SCFAs modulate the epigenetic status of genes that are expressed in neurons and investigate the role of SCFAs in enteric neurogenesis.

As microbe-derived molecules, formyl peptides, D-glycero-β-D-manno-heptose-1,7-biphosphate (HBP), and hydrocarbon receptor ligands (AHR ligands) are also necessary for enteric immune homeostasis. Therefore, it is expected that these molecules may have an effect on ENS development.

#### Enteric Endocrine System

As a neurotransmitter or hormone, 5-HT (also known as serotonin) modulates intestinal secretion and motility. Approximately 90% of the body’s 5-HT is produced by enterochromaffin cells (ECCs), which are a type of enteroendocrine cell that are present in the epithelium of the gut. 5-HT is synthesized by the rate-limiting enzyme tryptophan hydroxylase 1 (TPH1), before being stored in secretory granules. TPH2 also produces a much smaller fraction of the total 5-HT in the neurons of the myenteric plexus (Walther et al., 2003). Reigstad et al. (2015) observed that SCFAs, but not LPS, can promote TPH1 transcription in human ECCs. In turn, the activation of both submucosal and myenteric 5-HT4R-expressing neurons has been noted (Yano et al., 2015). Thus, gut bacteria that act through SCFAs are essential determinants of enteric 5-HT production and homeostasis, which in turn both affect the activity of the ENS. In addition to being biosynthesized, extracellular 5-HT within the gut is also regulated by serotonin-selective reuptake transporter (SERT), which is expressed in intestinal epithelial cells (IECs) (Bian et al., 2007) and is regulated by microbe-derived factors, such as the TLR ligand LPS (Bian et al., 2007).

Gut microbes regulate both bile acid synthesis and the production of secondary bile acids (Bian et al., 2007; Tremaroli and Bäckhed, 2012). Secondary bile acids can activate TGR5 (also known as protein-coupled bile acid receptor 1, GPBAR1), which is highly expressed in enteric neurons and enteroendocrine L cells (Poole et al., 2010). TGR5 signaling has been suggested to play a vital role in intestinal propulsive activity; the TGR5-dependent enhancement of peristalsis may also be partly mediated by the production of 5-HT (Alemi et al., 2013).

Other than 5-HT, SCFAs can also directly stimulate enteroendocrine cells [ECCs or enteroendocrine L cells (Panaro et al., 2014)] to produce several neuropeptides, such as peptide YY (PYY), neuropeptide Y (NPY), cholecystokinin, glucagon-like peptide-1 (GLP1), GLP2, gastric inhibitory polypeptide (GIP), and substance P (SP); these neuropeptides can influence GI functions and affect the development of the ENS.

#### Enteric Stromal Cells

The ENS not only comprises a broad range of neuronal subtypes but also harbors enteric stromal cells, including EGCs and ICCs. ICCs are fibroblast-like interstitial cells that regulate the pattern and frequency of peristaltic contractions (Sanders et al., 2014); they act as pacemakers in the vicinity of smooth muscle cells.

Enteric glial cells differ significantly from the Schwann cells seen in the PNS in that they do not form basal laminae. Furthermore, they show many similarities to CNS glia, in both morphology and function. Unlike enteric neurons, whose cell bodies are restricted to the ganglia in the muscularis propria and submucosa, EGCs are distributed throughout the gut wall, including in the lamina propria of the mucosa. EGCs are associated with both submucosal and myenteric neurons, the terminals of which run to the epithelial basement membrane and blood capillaries. EGCs coordinate signal propagation between myenteric neurons and epithelial cells, thus regulating gut motility, as well as the secretory and absorptive functions of the intestinal epithelium. In addition, EGCs maintain the integrity of epithelial barrier; they also participate in the development of the ENS by releasing specific mediators, e.g., glial cell-derived neurotrophic factor (GDNF), and by transforming growth factor β1 (TGFβ1) (Neunlist et al., 2014).

Kabouridis et al. (2015) demonstrated that EGCs in the submucous microenvironment are highly dependent on stimulation by the commensal bacteria that regulate the initial colonization of EGCs. Moreover, the mechanisms that underlie the interplay between gut microbes and EGCs rely on TLRs, particularly on TLR2 and TLR4, which are expressed in EGCs. Indeed, GF mice have been observed to show a marked decrease in the density of mucosal EGCs; this decrease could be restored upon recolonization (Kabouridis et al., 2015). Furthermore, the expressions of GDNF and glial markers (e.g., GFAP and S100β) have been observed to be significantly reduced in the myenteric plexus of TLR2–/– mice, while the administration of GDNF can rescue the ENS deficit. Thus, in this context, the microbe-TLR2 pathway promotes the functional maturation of EGCs and affects the development and homeostasis of the ENS via mesenchyme-derived neurotrophic factors (Turco et al., 2014).

It has been suggested that the molecular phenotype of EGCs exhibits a certain degree of plasticity. However, it is currently unclear as to whether this apparent plasticity of EGCs is manifested in all stages of adult life (Boesmans et al., 2015; Nagy and Goldstein, 2017).

#### Epithelial Cells

As membrane vesicles from the GI microbiota, MVs facilitate the movement of signals into the intestinal microenvironment. A recent study has identified that MVs indirectly communicate with IPANs in the myenteric plexus through unknown signals generated in the epithelium (Al-Nedawi et al., 2015). In this study, *Lactobacillus rhamnosus* and MVs formed by *L. rhamnosus* increased the number of action potentials recorded in preparations with an intact epithelium. However, no effect was observed when MVs were directly applied to IPANs of the myenteric plexus. Therefore, it appears that mucosal elements from epithelial cells are required to transduce the MV effect to the ENS.

## Neurological Disorders of the Gut

As a “second brain,” the ENS could be affected by neurodegeneration and other systemic diseases, similarly to the CNS. In addition, abnormalities in the ENS have been associated with severe GI disorders such as Hirschsprung disease (HSCR), esophageal achalasia (EA), gastroparesis, and neuropathic intestinal pseudo-obstruction. They have also been linked to a series of systemic diseases, which can manifest as intestinal innervation deficiencies; they have also been found to correlate with extra-GI diseases.

Many animal studies have observed a correlation between host aging and senescence-like phenotypic changes in the ENS. Host aging-related ENS degeneration includes dystrophic or degenerating nerve fibers; the alteration of the morphology of the enteric ganglia; and the accumulation of lipofuscin α-synuclein, hyperphosphorylated tau protein, and reactive oxygen species in aged enteric neurons (Thrasivoulou et al., 2006; Abalo et al., 2007; Phillips et al., 2009; Gamage et al., 2013). Aging-related alterations of the gut microbiota may instigate intestinal inflammation, which may eventually result in above aging-related ENS degeneration. Moreover, elderly and younger populations have shown differences in the compositions of their gut microbes (Maynard and Weinkove, 2018). It is inferred that the hallmarks of successful aging may be a balance among core microbiota as well as between pro- and anti-inflammatory activity (Badal et al., 2020).

### Deficiency of Intestinal Innervation

In the ENS, a complicated and timed migration of enteric neural crest derived progenitor cells leads to well-developed and interconnected networks within the gut wall. A lack of migration or differentiation of these enteric neural crest derived cells can result in the aganglionic innervation of the GI tract, which manifests as HSCR or EA.

The premature differentiation of progenitors into neurons or glial cells impairs the colonization of neural crest-derived cells in the distal bowel, resulting in missing enteric neurons at variable lengths in the intestine. Therefore, patients with HSCR exhibit intestinal obstructions. Patients with HSCR also exhibit altered gut microbial compositions, characterized by high levels of Bacteroidetes, Firmicutes, and Proteobacteria (Li et al., 2016). Pierre et al. (2014) demonstrated that a loss of the ENS results in defective intestinal motility, leading to intestinal bacterial overgrowth and increased abundances of pro-inflammatory bacterial lineages. Hirschsprung-associated enterocolitis (HAEC) has been shown to be closely related to the disturbance of the intestinal bacteria; the most abundant phylum detected in patients with HAEC and HAEC remission (HAEC-R) is Proteobacteria, followed by Firmicutes (Li et al., 2016). Additionally, Li et al. (2016) also observed that in each patient with HSCR, specimens from different intestinal sites differed significantly, but were more similar in each patient with HAEC and HAEC-R. Hirschsprung disease occurs through a complex process that involves multiple genetic mutations and disorders of the intestinal microenvironment. The causality that impacts the development of the ENS by gut microbes thus needs to be further investigated regarding the pathogenesis of Hirschsprung disease.

Esophageal achalasia is a motility disorder that is characterized by the failure of the lower esophageal sphincter (LES) to relax, and by esophageal dysmotility. The pathogenesis of neurogenic theory is widely accepted; it involves the selective degeneration of inhibitory neurons in the esophageal myenteric plexus (Pressman and Behar, 2017). The etiology of EA currently remains elusive, but several factors have been mentioned, including genetic predisposition, viral infection, and autoimmunity. Viral infections in individuals with a genetic predisposition may induce autoimmunity and the degeneration of inhibitory neurons in the esophageal myenteric plexus. Thus, viral infections may play an essential role in the pathogenesis of EA. However, more studies are needed in this regard.

### Correlation With Neurological or Systemic Diseases

Gastrointestinal function can be compromised by neurological diseases of the CNS, or by neuronal deficits induced by systemic diseases. For example, patients with stroke, Parkinson’s disease (PD), multiple sclerosis (MS), Guillain-Barré syndrome (GBS), amyotrophic lateral sclerosis (ALS), myasthenia gravis (MG), and other neurological diseases of the CNS often also experience digestive symptoms such as neurogenic dysphagia, abdominal distention, and constipation. These symptoms may arise from defects in any component of the MGB axis. Although neurological diseases in the CNS are considered as a potential influence on the GI tract, the underlying mechanisms still need to be studied further.

The ENS is vulnerable to neurological or systemic diseases. GI function may also contribute to neurological disorders inside or outside of the gut. GI dysfunction, e.g., malabsorption, celiac disease, ulcerative colitis (UC), and Crohn’s disease (CD), may lead to neurological symptoms. For example, the malabsorption of nicotinamide leads to neurological deficits and dementia (Fricker et al., 2018), while celiac disease has been shown to lead to neurological manifestations in up to 10% of all patients. The most common neurological symptoms are cerebellar ataxia, MS, peripheral neuropathy (PN), and other less described clinical conditions (Casella et al., 2016). PN in the form of ataxic and inflammatory demyelinating polyneuropathy has been found in inflammatory bowel disease (IBD) but is usually more severe in CD (Gondim Fde et al., 2015).

## Neurodegenerative Diseases in the CNS

Gut microbe-derived metabolites (e.g., SCFAs) can stimulate ECCs to produce neuropeptides or neurotransmitters, which, in turn, can diffuse into the bloodstream and reach the CNS. Besides, microbe-derived components can activate intestinal or circulating immune cells that can migrate to the CNS via the brain lymphatic network (Louveau et al., 2015; Rooks and Garrett, 2016). In addition, gut microbes also impact the ENS-vagus pathway (Furness, 2012). Therefore, bacterial molecules and neural-immune-endocrine pathways present a dynamic communication network in the MGB axis.

Short chain fatty acids produced by gut microbes ensure the integrity of the blood–brain barrier (BBB) by upregulating tight junction proteins; they also regulate the maturation and activation of microglial cells (Erny et al., 2015; Gao et al., 2021). In addition, SCFAs have also been implicated in the alleviation of stress-related disorders (van de Wouw et al., 2018). These examples reinforce the role of gut microbes in the MGB axis. Thus, it is reasonable to consider that part of the etiology of neurodegenerative diseases of the CNS may involve gut microbes.

The human ENS system changes gradually with aging, accompanied by the occurrence of neurodegenerative diseases. PD is characterized by the selective degeneration of dopaminergic neurons in the substantia nigra and Lewy bodies (abnormal depositions of α-synuclein) in the surviving dopaminergic neurons; this results in motor symptoms. A high percentage of patients with PD are characterized by abnormal GI motility and constipation. In fact, GI symptoms often appear before motor symptoms. Lewy bodies have been observed in the enteric neurons of patients with PD, which precede the development of motor symptoms by several years (Braak and Del Tredici, 2008). The ENS has been suggested to be an initial site of α-synuclein aggregations; these aggregations can subsequently spread to the brain through vagus nerve fibers. Indeed, patients undergoing vagotomies exhibit a reduced risk of developing PD (Svensson et al., 2015).

Additionally, Scheperjans et al. (2015) observed that the relative abundance of Enterobacteriaceae was positively associated with the severity of postural instability and gait difficulty, suggesting that intestinal bacteria are altered in PD, and are related to motor phenotypes. GFAP has been demonstrated to be overexpressed and hypophosphorylated in the EGCs of patients with PD, compared to those of healthy subjects and patients with atypical parkinsonism (Clairembault et al., 2014). Thus, EGC dysregulation might be associated with PD development. Besides, it has been found that shifts of the phage/bacteria ratio in lactic acid bacteria (produce dopamine and regulate intestinal permeability) are major factors in PD pathogenesis (Tetz and Tetz, 2018). The depletion of Lactococcus spp. in the PD patients was most likely due to the increase of lytic c2-like and 936-like lactococcal phages (Tetz et al., 2018). According to these studies, intestinal pathology could be an early and potential biomarker of PD. Nevertheless, it remains unclear as to whether the changes observed in gut microbiota contribute to the pathogenesis or consequences of PD.

In contrast with PD, few studies have investigated the enteric manifestations of Alzheimer’s disease (AD), despite it being the most common neurodegenerative disorder. AD is characterized by Aβ plaques and hyperphosphorylated tau protein at various stages, and ultimately in widespread cortical regions. A variety of taxa have been observed to be altered in patients with AD, with decreased levels of Firmicutes and Bifidobacterium, and increased levels of Bacteroidetes (Vogt et al., 2017). These alterations in gut microbes have been shown to correlate strongly with the pathological loads of Aβ and phosphorylated tau protein in patients with AD. Harach et al. observed that Aβ pathology was markedly diminished in GF mice with AD mutations (APP/PS1 model) in comparison to conventional AD animals (Harach et al., 2017). Moreover, early antibiotic treatment in AD mice has been shown to reduce Aβ deposition later in life (Minter et al., 2017). In addition, the treatment of AD mice with a *Bifidobacterium breve* strain can prevent Aβ-induced cognitive deficits, partially restore memory function, and improve inflammatory status (Kobayashi et al., 2017). However, another study showed no improvement in cognition for patients with severe AD after the consumption of a multistrain probiotic (containing *Lactobacillus* and *Bifidobacterium* strains) for 12 weeks (Agahi et al., 2018). Therefore, the translational value of using therapeutics to target the MGB axis for patients with AD remains unclear.

Huntington’s disease is a hereditary neurodegenerative disease that can cause progressive motor decline, cognitive dysfunction, and neuropsychiatric symptoms (Walker, 2007). Patients with Huntington’s disease also suffer from a range of GI disturbances, including diarrhea, nutrient deficiencies, and unintended weight loss (Djoussé et al., 2002; van der Burg et al., 2017). Kong et al. (2020) observed gut dysbiosis in R6/1 transgenic mice (a Huntington’s disease model), which was associated with body weight impairment and motor deficits.

Autism spectrum disorder (ASD) is one of the most common neurodevelopmental disorders worldwide (Maenner et al., 2020). ASD is characterized by stereotypical behaviors and deficits in social communication. In addition, patients with ASD often experience GI problems, which tend to correlate strongly with the severity of autism, and with increased irritability, anxiety, and social withdrawal (Gorrindo et al., 2012). Patients with autism have been reported to exhibit altered gut microbial diversity and increased abundances of *Clostridia* and *Candida* (Li et al., 2017; Luna et al., 2017). Animal models of ASD have also shown dysbiosis (Sgritta et al., 2019). Moreover, social impairments in these models were restored via the manipulation of microbiota (Hsiao et al., 2013; Sgritta et al., 2019).

Intestinal microbes are known to impact the development of the CNS through the brain-gut axis; they may even modulate it throughout the host’s life span. Further exploration of the modulation of gut microbiota and associated metabolic dysfunction may lead to the development of novel therapeutic approaches, such as enviromimetics, for a wide range of neurological and psychiatric disorders (Gubert et al., 2020).

## Prospective and Conclusion

As the second-largest genome in the human body, the intestinal microbiota have been the focus of significant attention regarding their regulation of the host and the external environment. New target molecules from the microbiota have been found and applied in practice. Despite a growing body of research, the MGB axis remains an obstacle regarding our understanding of its causal relationship, and regarding how the intestinal microbiota play a regulatory role in the systemic ENS, beyond the local intestinal tract. The ENS is a vital, irreplaceable bridge that connects intestinal microbiota with multiple systems. This review identified the following three aspects as being worthy of further study: (1) exploring how the microbiota act on the intestinal nervous system and its mechanism; (2) determining how the intestinal nervous system regulates intestinal function, thus affecting the intestinal microbiota; and (3) exploring how intestinal nerves regulate the physiological function of other nervous systems (such as the CNS). Meanwhile, it is noteworthy that multiple species differences should be carefully considered when applying findings from mouse ENS research to human GI studies (May-Zhang et al., 2021).

An accumulating amount of evidence suggests that bacterial molecules can pass the intestinal epithelial barrier to interact with enteric plexuses directly; they can also act on non-neuronal intermediary cells, whose products can be detected by enteric neurons. Gut microbes may also be involved in the development and maintenance of the CNS. Extensive metagenomic studies have determined that neurological diseases of the gut and brain are associated with the alteration of intestinal bacteria. Prebiotics, probiotics, and fecal microbiota transplantations (FMTs) have been used to manage certain neurological disorders to modulate gut microbes. In addition, increasing numbers of clinical trials have suggested that such treatments can provide beneficial effects to afflictions such as UC and *Clostridium difficile* infections (Paramsothy et al., 2017, 2019; Weingarden and Vaughn, 2017; Costello et al., 2019). Moreover, the therapeutic potential of microbiota-targeted interventions has also been emphasized in anti-aging medicine (Vaiserman et al., 2017).

**FIGURE 1:**
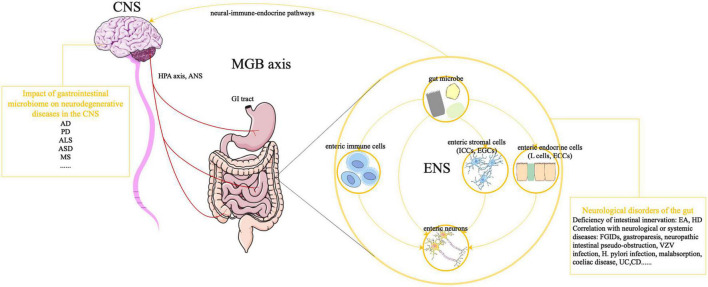
Effects of GI microbiota on the ENS and MGB axis. The ANS, in combination with the HPA axis and neuroendocrine signaling, can induce CNS-modulated changes in the gut. Meanwhile, bacterial molecules and neural-immune-endocrine pathways impact the development of the CNS, giving rise to a dynamic communication network in the MGB axis. The development and function of the ENS are controlled by GI microbiota, through direct or indirect mechanisms. Bacterial molecules can pass through the intestinal epithelial barrier to directly interact with enteric plexuses or act on non-neuronal intermediary cells (e.g., enteric immune cells, ICCs, EGCs, EGCs, and enteric L cells), whose products can be detected by enteric neurons. Abnormalities in the ENS are associated with life-threatening GI disorders, which can be manifested as intestinal innervation deficiencies (e.g., EA, HSCR) and can be correlated with neurological or systemic diseases (gastroparesis and neuropathic intestinal pseudo-obstruction). In addition, gut microbes might be associated with neurodegenerative CNS diseases, such as AD, PD, ALS, ASD, MS, and other neurological diseases. CNS, central nervous system; ANS, autonomic nervous system; HPA axis, hypothalamic-pituitary-adrenal axis; MGB axis, microbiota-gut-brain axis; AD, Alzheimer’s disease; PD, Parkinson’s disease, ALS, amyotrophic lateral sclerosis; ASD, autism spectrum disorder; MS, multiple sclerosis; GI, gastrointestinal; ENS, enteric nervous system; ICCs, interstitial cells of Cajal; EGCs, enteric glial cells; ECCs, enterochromaffin cells; EA, esophageal achalasia; HSCR, Hirschsprung disease; FGIDs, functional gastrointestinal disorders; VZV, varicella-zoster virus; *H. pylori*, *Helicobacter pylori*; UC, ulcerative colitis; CD, Crohn’s disease.

Therefore, in the future it will be essential to shift from simply conductive correlative analyses, and instead move toward prospective longitudinal studies, causative and mechanistic analyses, and larger scale trials of potential therapeutic approaches. Understanding the underlying mechanisms of gut microbe-ENS interactions could help to generate novel therapeutic applications for microbes regarding neurodegenerative diseases (Figure 1).

## Author Contributions

P-HZ, CZ, and Q-LL created the concept and designed the project. Z-HG and YZ drafted the manuscript. All authors contributed substantially to all aspects of the article and revised versions.

## Conflict of Interest

The authors declare that the research was conducted in the absence of any commercial or financial relationships that could be construed as a potential conflict of interest.

## Publisher’s Note

All claims expressed in this article are solely those of the authors and do not necessarily represent those of their affiliated organizations, or those of the publisher, the editors and the reviewers. Any product that may be evaluated in this article, or claim that may be made by its manufacturer, is not guaranteed or endorsed by the publisher.

## Abbreviations

5-HT: 5-hydroxytryptamine (or serotonin)
AChE: acetylcholine-esterase
AD: Alzheimer’s disease
Adrb2: encoding b2 adrenergic receptors [AR]
AHR ligands: hydrocarbon receptor ligands
ALS: amyotrophic lateral sclerosis
ANS: autonomic nervous system
ASD: autism spectrum disorder
BBB: blood brain barrier
BMP2: bone morphogenetic protein 2
BMPR: BMP receptor
CagA: cytotoxin -associated gene A
CD: Crohn disease
CNS: central nervous system
CSF1: colony stimulatory factor1
DCs: dendritic cells
DRG: dorsal root ganglia
EA: esophageal achalasia
ECCs: enterochromaffin cells
EGCs: enteric glial cells
ENS: enteric nervous system
FD: functional dyspepsia
FFAR2 (GPR43): free fatty acid receptor 2
FFAR3 (GPR41): free fatty acid receptor 3
FGIDs: functional gastrointestinal disorders
FMT: fecal microbiota transplantation
GBS: Guillain-Barré syndrome
GDNF: glial cell-derived neurotrophic factor
GF: germfree
GI: gastrointestinal
GIP: gastric inhibitory polypeptide
GLP1: glucagon-like peptide-1
GPBAR1: protein-coupled bile acid receptor 1 (TGR5)
*H. pylori*: *Helicobacter pylori*
HAEC: Hirschsprung-associated enterocolitis
HBP: D-glycero - β -D-manno-heptose-1,7-biphosphate
HSCR: Hirschsprung disease
HDACs: histone deacetylases
HPA axis: hypothalamic-pituitary-adrenal axis
IBS: irritable bowel syndrome
ICCs: interstitial cells of Cajal
IECs: intestinal epithelial cells
IPANs: intrinsic primary afferent neurons
LpMs: lamina propria macrophages
LPS: lipopolysaccharide
MAMPs: microbe-associated molecular patterns
MG: myasthenia gravis
MGB axis: microbiota-gut-brain axis
MMs: muscularis macrophages
MS: multiple sclerosis
MVs: microvesicles
MyD88: myeloid differentiation primary response 88
NE: norepinephrine
NPY: neuropeptide Y
NTS: nucleus tractus solitarius
PD: Parkinson’s disease
PGN: peptidoglycan
PN: peripheral neuropathy
PNS: peripheral nervous system
PRRs: pattern-recognition receptors
PSA: polysaccharide A
PYY: peptide YY
RSD: resistant starch diet
SERT: serotonin-selective reuptake transporter
SP: substance P
TGF β 1: transforming growth factor β 1
TLRs: toll-like receptors
TPH1: tryptophan hydroxylase 1
UC: ulcerative colitis
VacA: vacuolating cytotoxin A
VZV: varicella-zoster virus.
